# Exploring patients' experience of peer-supported open dialogue and standard care following a mental health crisis: qualitative 3-month follow-up study

**DOI:** 10.1192/bjo.2022.542

**Published:** 2022-07-22

**Authors:** Sailaa Sunthararajah, Katherine Clarke, Russell Razzaque, Marta Chmielowska, Benjamin Brandrett, Stephen Pilling

**Affiliations:** Institute of Psychiatry, Psychology and Neuroscience, King's College London, UK; Research and Development, Goodmayes Hospital, North East London NHS Foundation Trust, Goodmayes, UK; and Department of Clinical, Educational and Health Psychology, University College London, UK; Department of Clinical, Educational and Health Psychology, University College London, UK; Research and Development, Goodmayes Hospital, North East London NHS Foundation Trust, Goodmayes, UK; Research and Development, Goodmayes Hospital, North East London NHS Foundation Trust, Goodmayes, UK; and Department of Clinical, Educational and Health Psychology, University College London, UK; Department of Clinical, Educational and Health Psychology, University College London, UK; and Institute of Health and Wellbeing, University of Glasgow, UK

**Keywords:** Qualitative research, patients, crisis care, open dialogue, mental health services

## Abstract

**Background:**

Experience of crisis care may vary across different care models.

**Aims:**

To explore the experience of care in standard care and ‘open dialogue’ (a peer-supported community service focused on open dialogue and involving social networks for adults with a recent mental health crisis) 3 months after a crisis.

**Method:**

We conducted semi-structured interviews with 11 participants (6 received open dialogue; 5 received treatment as usual (TAU)) in a feasibility study of open dialogue and analysed the data using a three-step inductive thematic analysis to identify themes that (a) were frequently endorsed and (b) represented the experiences of all participants.

**Results:**

Four themes emerged: (a) feeling able to rely on and access mental health services; (b) supportive and understanding family and friends; (c) having a choice and a voice; and (d) confusion and making sense of experiences. Generally, there was a divergence in experience across the two care models. Open dialogue participants often felt able to rely on and access services and involve their family and friends in their care. TAU participants described a need to rely on services and difficulty when it was not met, needing family and friends for support and wanting them to be more involved in their care. Some participants across both care models experienced confusion after a crisis and described benefits of sense-making.

**Conclusions:**

Understanding crisis care experiences across different care models can inform service development in crisis and continuing mental healthcare services.

Care after a mental health crisis varies across different countries and care models, resulting in varying experiences. A ‘crisis’ can be defined as a reoccurrence or increase in the severity of clinical symptoms of a mental disorder, often occurring as a response to difficult personal circumstances, such as relationship problems or financial uncertainty.^1^ In the UK, the definition or determination of what constitutes a crisis can vary across mental health services, although measures such the UK Mental Health Triage Scale^2^ have been developed to aid in such decisions. In the UK, a crisis associated with a new mental health problem or the relapse of an existing problem is cared for by the National Health Service (NHS) and often requires the involvement of a community mental health team (CMHT), crisis resolution and home treatment team (CRHT) or in-patient service.^3^ In-patient care is necessary when the level of risk or support needed cannot be effectively managed in the community. The National Institute for Health and Care Excellence highlights that individuals in crisis should have access to a 24-h helpline linking them to a CRHT, which should conduct an assessment within 4 h of the call to determine how their care will be managed.^4^ However, the assessment can also come from a general practitioner (GP) or other services who can identify a crisis and triage to the appropriate service.^4^

At present, CRHTs are the specialist service for crisis care, showing positive evidence on clinical and cost-effectiveness, although greatly varying in implementation.^5^ Currently, there is a lack of evidence examining the efficacy of other services in crisis care, although there is general evidence of benefit from such services for individuals with severe mental health problems. For example, a review found that, compared with standard non-team management, CMHT care for people with severe mental health difficulties was associated with lower hospital admissions and less non-satisfaction with services.^6^ In-patient care is also associated with improved functioning.^7^

Experiences of NHS crisis and community mental healthcare are mixed. In 2019, the Care Quality Commission conducted a survey of patients of CMHTs in which most respondents (80%) reported that they received the care they needed during a crisis – but 20% said that they did not.^8^ Although most respondents knew who to contact in the NHS out of hours if they had a crisis, nearly one-third (31%) did not.^8^

A qualitative study of focus groups and interviews exploring patient and carer experience of CRHTs indicated that services were often experienced as prioritising medication rather than providing emotional and practical support, a range of interventions or involving patients’ social networks.^9^ Patients also found the lack of staff continuity problematic and carers often reported feeling excluded. However, services’ rapid initial response and frequent home visits were valued and seen as central to good care.

Experiences of in-patient services have often included reports of worsening clinical symptoms, restricted freedom and relationships that are alienating but they are also sometimes acknowledged as necessary and the best thing for them at the time.^10,11^

## Open dialogue

To address these concerns and others, for example continuity of care, ‘open dialogue’ services have been developed which aim to combine crisis and continuing mental healthcare into one service, in contrast to a functional team model adopted by many mental health services in England.^12^ Early versions of open dialogue were influenced by the needs-adapted approach to treatment, as in the original Finnish projects.^13^ The Western Lapland research participants were interviewed approximately 19 years after their treatment using an open dialogue-based approach and generally they did not provide specific comments on open dialogue or any other specific techniques for improving their mental health, perhaps because the open dialogue approach was not a novel way of working in the region, but they emphasised their own actions, changing living situations, social relationships and so on as contributing towards change.^14^ However, when asked further about their treatment experiences, they viewed network meetings as mainly positive as they enabled interaction with other people and the chance to go through difficult experiences. Further, family involvement was regarded as a mainly positive or neutral factor and a minority had mixed experiences regarding their treatment, including some characteristic features of open dialogue.

Open dialogue is an integrative approach that embodies systemic family therapy^15^ and has growing interest internationally.^12,16–20^ Some note that open dialogue's increasing popularity is due to its compatibility with a human-rights approach.^21^ Key aspects of open dialogue include continuity of care, immediate care, tolerance of uncertainty, dialogic practice and clinical meetings that involve patients’ networks.^12,22–24^ A core feature of the open dialogue model is valuing individuals with ‘lived experience’ and therefore peer support workers are integral members of the clinical team.^12^

## The evidence base

A recent review of open dialogue^25^ found that there is presently a lack of methodologically rigorous quantitative studies (e.g. owing to high heterogeneity in the models of open dialogue and outcome measures utilised), and therefore conclusions on efficacy are limited. The review also found qualitative studies of open dialogue to be of low quality and at high risk of bias.

Nevertheless, over the past 2 years, emerging qualitative approaches have looked to understand the experience of open dialogue from a patient perspective. Research suggests that patients usually reflected positively on their experiences of open dialogue when considering previous encounters with treatment as usual (TAU).^17,26,27^ However, these studies did not directly explore the experiences of both open dialogue and TAU within the same analyses. There is minimal evidence comparing the experiences of open dialogue and TAU. Piippo (2008) conducted interviews with people who had previously experienced TAU and more recently open dialogue and the latter was experienced as positive, negative and ambivalent, although involving relatives in meetings created an experience of safety for individuals from all sides.^28^ Further research is required to better understand what determined such experiences.

Wusinich et al (2020) reported that patients who had experienced open dialogue valued the accessibility of services and the dilution of role hierarchies, but attitudes towards medication management and the structure of network meetings seemed mixed.^19^ Tribe et al (2019) found that patients had ‘mixed’ experiences of open dialogue: although most felt listened to, some found clinicians’ reflections (an integral aspect of the model) unusual and one found it distressing.^23^ Hendy et al (2019) used a series of focus groups to explore patients’ experiences of open dialogue; participants reported being viewed as equal in network meetings, being able to relate to practitioners and also commented on the value of decisions being made together.^29^ Recent further qualitative work has also examined the experiences of practitioners implementing open dialogue. Current evidence highlights the institutional and contextual constraints of full integration of open dialogue as well as resistance in the shifting of professional identity paradigms that open dialogue brings.^30,31^

‘Open dialogue: development and evaluation of a social network intervention for people with severe mental illness’ (ODDESSI) is large multi-site cluster randomised controlled trial currently underway, mainly exploring the clinical and cost-effectiveness of open dialogue compared with TAU in people presenting to mental health services with a crisis. The ODDESSI trial includes a feasibility study, a main study and various elements of qualitative work exploring experiences of care. The study presented in this paper was undertaken as part of the feasibility trial. It aims to explore the experiences of a group of these participants 3 months after presenting in crisis to services, as there is currently limited evidence exploring experiences of open dialogue and TAU.

## Method

### Open dialogue and TAU

The open dialogue participants in our sample were under the care of the open dialogue team and their treatment comprised regular network meetings based on the open dialogue model, which includes medication reviews by the team's psychiatrist. The open dialogue staff all undergo 4 weeks’ training in the approach across the year while undertaking clinical placement in an open dialogue service. The urgency of participants’ crises was categorised on the UK Mental Health Triage Scale, and all participants had presented with a crisis meeting criteria A, B or C.^2^ The TAU participants’ care experiences varied: three were under the care of their GP (one individual's treatment mainly comprised medication review; the other two did not regularly seek support from their GP and therefore were not actively receiving any treatment), although one of them was initially assessed and briefly seen by a CRHT; one was under the care of secondary care psychological services, receiving specialist support for depression and anxiety (although previously had been under the care of a CMHT); and one individual was on the waiting list for Improving Access to Psychological Therapies services and was not actively receiving any care, although had been initially assessed and briefly seen by a CRHT. TAU staff did not have any additional/specialist training for the purpose of the ODDESSI trial. There were no fidelity measures for either group.

### Sample

The ODDESSI feasibility study ran from June 2018 to May 2019; 60 participants were recruited across two NHS sites in England. Owing to limited resources, the current study is based on only one of the sites, which is located in a suburban area in outer London, where 29 participants had been recruited. All 29 were contacted by telephone to see whether they would be willing to take part in a study that aimed to understand their experiences of their care. Eighteen of these individuals did not take part: most were no longer reachable via their contact details (*n* = 12), some declined (*n* = 5) to participate (as they were busy) and one had withdrawn from the ODDESSI study (no reason given). In total, 11 individuals (6 had received open dialogue; 5 had received TAU) agreed to participate and were invited to the interview.

### Interviews

Individual interviews were conducted by two female researchers (S.S. and M.C.). The positions of both researchers and all authors on this paper should be noted: all authors were involved with the ODDESSI trial and are therefore influenced by their understanding of open dialogue. Both researchers had prior experience in conducting interviews for qualitative research. The interviewees understood that the researchers were research assistants on the ODDESSI trial and their aim was to gather an understanding of the interviewees’ care. The interviews were conducted at a location convenient for the participant. This was either their home or a local NHS mental health clinic. No one besides the researcher and participant was present in the interviews. All participants were asked for permission to audio-record their interviews. The average duration of the interviews was 30–45 min. An interview schedule was developed to explore patient experiences of care. A topic guide was developed drawing on prior research and the early stages of the ODDESSI study (see Appendix below). The topic guide was not pilot tested. A semi-structured format was adopted in which follow-up prompts were often used to encourage participants to expand on their answers.

### Data analysis

All audio files were transcribed by a professional transcription company. Three researchers were involved in the data analysis (S.S., M.C. and K.C.). Two researchers (S.S. and M.C.) independently used a six-step inductive thematic analysis process^32^ as a framework to analyse transcripts. First, they familiarised themselves with the transcripts and generated initial codes. In a collaborative and reflexive process, codes were then developed into broader themes which looked to capture the experience of the participants. Both S.S. and M.C. met frequently to discuss initial interpretations, generate themes, sense-check and progressively refine generated themes. They also looked to understand the commonalities held within participant narratives. Throughout this process, a third author (K.C.) was involved in discussions on the rationale of themes and resolved any conflicting interpretations or disagreements, and a fourth author (B.B.) encouraged the team's reflexivity and for the codes and themes generated to be understood through the lens of analysts who had unique experience of open dialogue. The themes were then refined and clear descriptions were developed to reflect the broader meanings of the participant narratives.

### Data presentation

The identified themes are presented using headings that contain participants’ own words. All 11 participants are represented in the themes. If quotes within themes are frequently endorsed (either between or within treatment groups), this is highlighted. All participants were anonymised. If participants mentioned their clinicians’ names, it was removed and replaced with ‘[clinicians’ names]’. To ensure clarity for the reader, some things the participants refer to are explained in square brackets, for example, ‘it’ is explained as ‘[referring to treatment]’.

### Ethics

The authors assert that all procedures contributing to this work comply with the ethical standards of the relevant national and institutional committees on human experimentation and with the Helsinki Declaration of 1975, as revised in 2008. All procedures involving human participants were reviewed by the National Institute for Health Research and approved by the Bromley Research Ethics Committee (Ref: 18/LO/0868). Approval for the study was obtained from the Integrated Research Application System (IRAS): ID 233243. Written informed consent was obtained from all participants.

## Results

### Participant demographics

The 11 participants (6 male; 5 female) were aged between 26 and 58 years (mean 43; s.d. = 11.7) and all were White British; 6 were under open dialogue and 5 were under TAU.

### Themes

In the exploration of the open dialogue and TAU participants’ experience of NHS care following a mental health crisis, four main themes emerged that are highly represented across the participants. There is good representation of the themes across both groups, although two themes (‘having a choice and a voice’ and ‘confusion and making sense of experiences’) are more represented in the open dialogue group. Most themes are also represented by most participants in each group, aside from two themes (‘having a choice and a voice’ and ‘confusion and making sense of experiences’) in the TAU group.

Subthemes are included in each main theme that often encapsulate the different experiences of open dialogue and TAU participants.

#### Theme 1: Feeling able to rely on and access mental health services

Participants described feeling that it was important to have mental health services that they could rely on. Participants tended to discuss ease of access as a principal component in fostering reliance. Open dialogue participants, Participants A, B and E, for example, described feeling supported in the knowledge that help was available if needed and, as Participants B and E explained, the process of obtaining support was straightforward:

‘Thinking that you've got some kind of support out there’ (Participant A, open dialogue)

‘They've always been on the other end of the phone, I can text them and everything’ (Participant B, open dialogue)

‘I know that if I need help, I can just contact them’ (Participant E, open dialogue).

However, when this need was not met, people described exasperation and feeling ‘let down’ by services. This was more commonly experienced by the TAU participants. For example, some TAU participants described difficulty in accessing services and feeling particularly invalidated when attempts to obtain support from services were denied regardless of a voiced need. As a result, participants acknowledged disappointment and feeling abandoned:

‘I had a discussion with her and she said she would have to talk to her team and she would phone me back at four o'clock in the afternoon with the results of how they could help me. So, that telephone call came at four o'clock, she said that they felt they couldn't help me, that the Job Centre would be my best port of call and her parting words to me were, ‘Happy job-hunting’. I had just told that woman that I felt suicidal and that's what I got’ (Participant C, TAU)

‘And they started it [referring to treatment] and then the person [referring to clinician] left, so left me… It [referring to treatment] was just short, and the person [referring to clinician] weren't there to do it anymore, so they just left me in the lurch’ (Participant D, TAU).

#### Theme 2: Supportive and understanding family and friends

Participants indicated that having a supportive and understanding network of family and friends was integral to their mental health and well-being. Open dialogue participants and TAU participants contributed to this theme. Participants often valued the role of their family and friends in their clinical sessions as it helped build their own support system. For example:

‘most every session, one of my parents were with me, so they were able to elaborate things for me better’ (Participant A, open dialogue)

‘I've only involved two people which is the two that I live with. They're like an adopted mum and dad to me. I haven't involved anyone else because they just wouldn't understand. […] The sessions just got better and better, and it's allowed me to now be open about everything rather than keep it all inside. And it's allowed the family to see if I'm struggling, and know what to say to me and sort of try and help’ (Participant E, open dialogue).

However, the desire to involve family and friends in their care was not always there. For example, one participant felt uncomfortable with his wife seeing the impact his difficulties had on him during discussions with his clinicians. He felt that he did not want to involve his family in clinical sessions as he felt that his mental health problems should remain private:

‘You know, because some of the stuff I talk about in these sessions I don't even talk to my wife about. She knows about them, but I keep them to myself… I don't want her to see the state I get in sometimes when I talk about it, and then to have to have two other people [referring to clinicians] there as well, I just kind of … them conversations are for my private life, that's how I kind of felt about it’ (Participant J, open dialogue).

For the TAU participants, supportive and understanding family and friends were integral to their mental health and were seen as being there to pick up where services had not been available:

‘I do feel that the people you trust helped me a lot. They're the ones who forced things, pushed me, they came along to interviews with me, to see people and things like that’ (Participant C, TAU)

‘My husband, he was really good and my grandchildren are everything to me, so they helped … it's really hard to explain, it was my husband and my grandkids that got me through, I think, definitely. […] When I stop listening to music, my husband knows that I'm going to dip, so then he'll encourage me to put the music on and that helps a lot’ (Participant F, TAU).

However, one TAU participant acknowledged that, although she did have a network of friends and family, she felt that she was unable to engage with them outside of general conversation and found difficulty in opening up to them about her problems without them asking:

‘I have friends and family, but I wouldn't go to them with it and they don't ask me how I am so I wouldn't open up to them. […] Sometimes you need someone to give you a bit of motivation […] Like, I don't have anyone to bounce off of, I don't have anyone to talk to apart from general things like what do you want for dinner? Or I have put the rubbish out, that's about it, but no conversation, no one who sort of understands’ (Participant G, TAU).

#### Theme 3: Having a choice and a voice

Participants valued experiences of autonomy and having a choice and a voice in their treatment encounters (for example, in being able to discuss their mental health needs and be involved in their treatment plan). All open dialogue participants discussed the importance of this theme. Open dialogue participants valued their choice, for example, in having autonomy within sessions, which allowed for breaks when they felt they were needed:

‘I could say what I wanted, I wasn't judged and when actually I was getting upset, because I did cry quite a bit to be truthful, and I'm not embarrassed to say it, but it was like I was welcome to pop out and have a quick fag, no problems’ (Participant B, open dialogue).

Participants spoke of having a voice in treatment planning and acknowledged the benefits of a transparent and collaborative process:

‘Every decision made was with me, [clinicians’ names’]. It wasn't a case of them forcing anything on me whatsoever, we all agreed together’ (Participant E, open dialogue).

Some participants acknowledged the importance of having a choice; that is, a choice in being able to decide the frequency and location of meetings and a choice in how their supportive network were involved in sessions:

‘It's at my discretion how often we meet, and where we meet, and when we meet […] The being able to invite – whether it be my husband, or a friend, or my sisters, or anybody else within my social context – are the things that has I suppose, worked the best’ (Participant H, open dialogue)

‘From very early on I was always asked would I like to have home visits, would I like to bring my husband with me, my mum with me, any support I wanted to bring […] I was involved in all the discussions. Obviously open dialogue they discussed between themselves, but I was also included, I was asked are you okay with this, are you okay with that, would you like to be referred to someone as well as the medication, and … so, yeah, I felt very involved as well’ (Participant I, open dialogue).

Some TAU participants contributed to this theme, with one person mentioning their frustration in feeling that their voice was not being heard:

‘Like if I speak to her [referring to GP] and everything is “You have got this but it's not as bad as you think”. So why am I in so much pain then? They are saying it is not as bad as what you think, you're telling me how I feel’ (Participant G, TAU).

She also expressed her difficulty in having her voice heard as she had no choice over the duration of sessions:

‘when I go to the doctor, I find it hard to speak to her because you have to just hurry up basically’ (Participant G, TAU).

Another TAU participant was asked if he felt in control of his treatment and he said:

‘I didn't feel in control at all’ (Participant C, TAU).

#### Theme 4: Confusion and making sense of experiences

The majority of participants experienced some confusion as part of their mental health crisis, for example not knowing what was happening to them or what kind of care they would get. All open dialogue participants and some TAU participants contributed to this theme. The confusion and need to make sense of experiences was more frequently endorsed by open dialogue participants than TAU participants.

Several participants receiving open dialogue noted the collaborative experience of sense-making and feeling validated when medical professionals reflected using the participant's own words. For example:

‘Sometimes to hear the words that you may have said but may not have actually heard said back to you through someone else's voice is very powerful. To have that contextualised within a professional medical opinion, makes it resonate very strongly’ (Participant H, open dialogue)

‘there are things that happened in my childhood that has contributed towards it, and I didn't realise, I just sort of buried it for a very long time. And without forcing me to talk about it they made me just realise that there are issues there, and I felt better for like realising that’ (Participant I, open dialogue).

However, this was different for one person who received open dialogue, who felt that there was no meaning-making of his experiences and he addressed this frequently throughout the interview. For example:

‘I didn't really get any closer to what might be causing this, or why these feelings are happening, or why the ups and downs and the swings in my mood. I didn't get any closer to any kind of answer from it. […] I kind of felt like when I was going to go into stuff like this that people would help you get past the surface, you know, maybe help me understand what's actually happening to myself, and then maybe teach me some techniques or anything’ (Participant J, open dialogue).

Some TAU participants highlighted their confusion after their crisis and appeared angry and deflated over not having sense made of their experiences. For example:

‘I just wanted to get to the bottom of everything because I have never asked for the help before in that respect’ (Participant G, TAU)

‘it's frustrating not knowing when it's [referring to treatment] going to start, because obviously, I'm trying to get promotions at work, I'm trying to do a lot with my life and sort my life out for personal reasons. And not being able to do that in my current situation is very irritating like I have to double-guess and sometimes triple-guess what I'm doing’ (Participant K, TAU).

An open dialogue and a TAU participant remembered feeling confused about their care and treatment plan shortly after the crisis, with one (Participant K) speaking of the importance of communication after a crisis and the feelings of frustration that resulted when it was lacking:

‘I wasn't too sure what was going on in the beginning though […] I wasn't sure how long it [referring to treatment] would be or anything like that’ (Participant A, open dialogue)

‘I think, from the clinical point of view, there needs to be better communication. Obviously, I haven't heard anything in nearly six months, and that's a long time to wait. I need to know how far away from getting the treatment I am instead of just being left in the dark like I have been. Because that's probably the most frustrating part, I know I'm going to get the treatment eventually, but I don't know when’ (Participant K, TAU).

## Discussion

By exploring participants’ experiences of NHS care 3 months after a mental health crisis, the present study has identified four main themes signifying what is most important to them. Subthemes emerged encapsulating the different experiences of open dialogue and TAU participants.

‘Feeling able to rely on mental health services’ (theme 1) was the most common theme that emerged from the entire sample. Participants spoke of the importance of services being available and easily accessible after a mental health crisis. The importance of this theme was typically illustrated by participants describing either positive experiences where services were available and accessible after a crisis, or negative experiences where participants discussed a desire to engage with services but were denied access. Further to this, participants also discussed how ease of access was important, with open dialogue participants in particular speaking of the benefit of being able to contact the team directly if they were to re-engage.

‘Supportive and understanding family and friends’ (theme 2) was also a very common theme that emerged. However, the subthemes indicate that open dialogue participants and TAU participants valued a supportive and understanding family and friends in different ways. Open dialogue participants valued the significant contributions of family and friends in network meetings and the role they subsequently played in their clinical care. On the other hand, TAU participants felt the importance of having supportive and understanding family and friends during a difficult time after a mental health crisis, when mental health services may not have been easily accessible. Nonetheless, the emergence of this theme highlights the fundamental importance of having a support network for patients after a mental health crisis.

‘Having a choice and a voice’ (theme 3) was typically synonymous with the experience of open dialogue. However, both TAU and open dialogue participants discussed interactions with providers that were contextualised by either a presence or absence of autonomy. Open dialogue participants reflected on opportunities in having a voice and choices in their clinical care and the importance of these interactions. On the other hand, some TAU participants discussed limited opportunity to have a voice and choice in their clinical care as their needs may be minimised or not provided the time to properly discuss them. This theme highlights the importance of communication, collaboration, transparency and patient autonomy in clinical care and enabling patients to make decisions.

‘Confusion and making sense of my experiences’ (theme 4) was endorsed by both open dialogue and TAU participants, who described feelings of confusion about what was happening to them in terms of both their mental health and how their clinical care was going to be set out. It is important for this to be addressed and for clinical teams to ensure that there is meaning-making behind patients’ experiences and that care pathways are clearly outlined.

Similar to previous research, patients have mostly valued the benefits of the open dialogue service model,^29^ although experiences are ‘mixed’.^23^ For example, participant J highlighted how he did not like involving his social network in clinical meetings and how sense was not made of his experiences.

### Strengths and limitations

A key strength of this study is that it provides an in-depth exploration and understanding of individual experiences of open dialogue and TAU 3 months after a mental health crisis, thereby providing us with very rich information. However, qualitative studies are limited in some ways. First, the experiences described are those of a very small and particular sample and cannot be representative of a broader population; for example, those living in different areas might have different experiences based on care pathways. Further, some ideas are only expressed by a single participant (e.g. Participant J did not want to involve his family in his care and found open dialogue confusing). Although some of the information shared by individuals may not be highly representative, it is still very useful and important for open dialogue services. It is crucial to note that generalisations from this study should be made with caution owing to the very small sample size.

Further, many TAU participants were not directly receiving any specialist mental health support at the time of interviewing. It is therefore important to remember the vast range of care pathways after a mental health crisis which can determine experiences. This is a limitation related to the small sample size, as a bigger sample might have encompassed a greater range of models of care and thereby the experiences might have varied. The ODDESSI main trial will seek to address this limitation as it will comprise a larger and more representative sample of TAU.

It is also important to consider that there is currently great variability across CRHTs,^5^ reflecting a crucial limitation in the present study when understanding the experiences of participants who were initially under their care immediately after a crisis. The lack of a fidelity measure is also a limitation of the trial. This limitation will also be addressed in ODDESSI's main trial.

Another limitation of this qualitative approach is that experiences that are not frequently endorsed or not representative of most participants are missed, which might risk missing unique experiences. However, the risk of this problem is very low, as careful analysis ensured that we captured all individuals’ experiences in the themes. Nevertheless, the results indicate that there are two themes (‘having a choice and a voice’ and ‘confusion and making sense of experiences’) that are not highly represented in the TAU group.

A further limitation that should be noted is the lack of iterations with participants, which highlights that future research could maximise themes.

Lastly, both research assistants and the broader study team have an interest in open dialogue and, although a systematic, stepwise approach was taken in the development of themes, the position and experience of the study team should be considered in this analysis. That is, the study team are all involved in the implementation of ODDESSI and to a broad degree bring a comparative framework for understanding open dialogue within the wider context of NHS TAU.

### Future directions

It would be valuable to think about exploring patients’ experiences of NHS care on a more long-term basis, for example 6 months or a year after a mental health crisis, as experiences might change over time. As ODDESSI's main trial is currently underway, it would be valuable to investigate the experiences of these participants on a wider scale. It may be particularly valuable to further explore unique features of open dialogue and TAU models; for example, as regards the former, how participants experienced the support of a peer support worker. This could further decipher key strengths across differing care models.

## Data Availability

Data availability is not applicable to this article as no new data were created or analysed in this study.

## Appendix

### The topic guide

1. Can you describe your experience of the care you have received during your participation in the ODDESSI trial?
2. How did you feel about the care you received?
3. Did you feel in control of how your treatment was going and how it was going to turn out?
4. Were you happy with the extent others in your family/friends/social network were involved in your treatment?
5. Was there anything that was particularly helpful or worked well in your care – if so, can you elaborate?
6. Was there anything that was not helpful or didn't work well in the care you received – if so, can you elaborate?
7. What changes did you experience in yourself as a result the treatment you received?
8. Is there anything I haven't asked today that you think might be important or helpful for us to know?
9. Any final thoughts?
